# Copeptin—Marker of Acute Myocardial Infarction

**DOI:** 10.1007/s11883-014-0421-5

**Published:** 2014-05-21

**Authors:** Martin Möckel, Julia Searle

**Affiliations:** Department of Cardiology and Division of Emergency Medicine, Campus Viorchow Klinikum (CVK) and Campus Charité Mitte (CCM), Charité – Universitätsmedizin Berlin, Charitéplatz 1, Berlin, 10117 Germany

**Keywords:** Copeptin, Troponin, Rule-out, Acute myocardial infarction, Acute coronary syndrome

## Abstract

The concentration of copeptin, the C-terminal part of pro-arginine vasopressin, has been shown to increase early after acute and severe events. Owing to complementary pathophysiology and kinetics, the unspecific marker copeptin, in combination with highly cardio-specific troponin, has been evaluated as an early-rule-out strategy for acute myocardial infarction in patients presenting with signs and symptoms of acute coronary syndrome. Overall, most studies have reported a negative predictive value between 97 and 100 % for the diagnosis of acute myocardial infarction in low- to intermediate-risk patients with suspected acute coronary syndrome. Additionally, a recent multicenter, randomized process study, where patients who tested negative for copeptin and troponin were discharged from the emergency department, showed that the safety of the new process was comparable to that of the current standard process. Further interventional trials and data from registries are needed to ensure the effectiveness and patient benefit of the new strategy.

## Introduction

Ever since Reichlin et al. [1] published their article entitled “Incremental value of copeptin for rapid rule-out of acute myocardial infarction” in 2009, the concept of a single, combined troponin and copeptin test at admission in patients with suspected acute coronary syndrome (ACS) has been tested and reported in an increasing number of publications, with conflicting results and conclusions.

Copeptin is the C-terminal part of pro-arginine vasopressin. Vasopressin is an important hormone for water homeostasis, but has a very short half-life and is unstable in vitro, which makes its quantification difficult. The function of copeptin remains unknown, but it is secreted in equimolar quantities as vasopressin and has the advantage of high stability in blood samples.

Owing to its involvement in the ACTH cycle, copeptin has been proposed to be a marker of severe stress reactions on top of the hemodynamic triggers mentioned above. The data base for this assumption is scarce [2].

The pathophysiological model of acute myocardial infarction (AMI) rule-out is straightforward. Copeptin is an unspecific marker but its concentration increases early on an acute event such as AMI, most probably owing to the drop in cardiac output and/or blood pressure. Troponin, on the other hand, has 100 % cardio-specificity, but its concentration takes time to increase after myocardial necrosis has occurred. To ensure there is ischemia-related myocardial necrosis, the universal definition of AMI requires “a rise and/or fall of cardiac biomarker values [preferably cardiac troponin (cTn)] with at least one value above the 99th percentile upper reference limit (URL)” [3], and thus serial troponin measurements within a timeframe of 3–6 h. As a consequence, a positive test result for either marker at presentation cannot be used to confirm the diagnosis of AMI. The strength of the combined marker strategy with copeptin and troponin is the very early rule-out of myocardial infarction with a single blood draw, as copeptin and troponin should cover all time frames after the acute event.

This article summarizes the evidence on the added value of copeptin as a rule-out marker for AMI in patients with suspected ACS to evaluate whether this pathophysiological model holds true in clinical application.

## The Clinical Need

Acute chest pain is one of the most frequent chief complaints in internal emergency medicine. In the CHARITEM study, which analyzed data of 34,333 internal emergency patients of a German University hospital, 11.5 % (*n* = 3,954) of the patients presented with a leading symptom of chest pain. Of these, 47.5 % (*n* = 1,879) were admitted to the hospital, but only half of them where diagnosed with an ACS, and only 21.4 % (*n* = 402) had a final diagnosis of AMI. This means for the 3,954 patients presenting with chest pain, the frequency of AMI was as low as 10.2 % [4•]. In a study by Goodacre et al. [5] from the UK, 6 % (*n* = 6.957) of all emergency patients presented with chest pain, of which 11.0 % had ECG evidence of ACS and 34.5 % had clinically diagnosed ACS. In patients admitted to the hospital (*n* = 4,438), 16.1 % had ECG evidence of ACS and 48.6 % had clinically diagnosed ACS. In the USA, chest pain was the second leading reason (5.4 %) for emergency department (ED) visits in 2010. A principal hospital discharge diagnosis of ischemic heart disease was assigned to 2.9 % of all ED visits [6].

Even though not all chest pain patients will cause the treating physician to suspect ACS and even though ACS also needs to be suspected in many patients with symptoms other than chest pain, these numbers emphasize the clinical need for an early and fast rule-out of AMI. Under the current standard process of evaluating these patients, a minimum stay of between 3 and 9 h is mandatory. Given that the vast number of these stays are ultimately unnecessary, personnel and space resources are wasted in the ED and patients are left to wait until the possible diagnosis of a “heart attack” has dissolved. In times of increasing ED crowding, which has been shown to negatively impact patient outcome [7], this process is basically unacceptable.

## The Current Evidence

The two hallmark articles on copeptin in patients with suspected ACS were published by Reichlin et al. [1] and Keller et al. [8] (Table 1).

**Table 1:** Summary of the copeptin studies by Reichlin et al. [1] and Keller et al. [8]

Study	MI prevalence	Troponin assay	Copeptin assay	NPV^a^	Comments
Reichlin et al. [1]	ED population (single-center study). AMI 16.6 % (81/487). NSTEMI 10.5 % (51/487)	TnT (Roche Diagnostics, Germany) cutoff 0.01 μg/l	Copeptin KRYPTOR (Thermo Fisher B·R·A·H·M·S) cutoff 14 pmol/l	AMI 99.7 %	No NPV calculated for NSTEMI alone. At presentation, 25 % of patients were troponin-negative
Keller et al. [8]	CPU population (3 study sites). AMI 21.6 % (299/1,386). NSTEMI 14.9 % (206/1,368)	TnI-Ultra (Siemens Healthcare) 40 ng/l (99th percentile) (all sites)	Copeptin KRYPTOR (Thermo Fisher B·R·A·H·M·S)	AMI:	37.3 % of patients presented within 3 h after symptom onset; 58.2 % presented within 6 h after symptom onset
			9.8 pmol/l (95th percentile)	99.0 % (96.6–99.9 %)	
			13 pmol/l (97.5th percentile)	98.3 % (95.6–99.5 %)	
			18.9 pmol/l (99th percentile)	98.4 % (96.1–99.6 %)	
		TnT (Roche Diagnostics) cutoff 30 ng/l (2 sites)	9.8 pmol/l	95.8 % (93.9–97.2 %)	NSTEMI patients only: TnT plus copeptin (9.8 pmol/l) NPV 96.5 %
			13 pmol/l	94.8 % (93–96.3 %)	
			18.9 pmol/l	94.1 % (92.2–95.7 %)	

*AMI* acute myocardial infarction, *CPU* chest pain unit, *ED* emergency department, *MI* myocardial infarction, *NPV* negative predictive value, *NSTEMI* non-ST-segment-elevation myocardial infarction, *TnT* troponin T

^a^NPV for marker combination if not indicated otherwise.

Reichlin et al. [1] first hypothesized “that the combination of a marker of cardiac necrosis, such as troponin, with a pathophysiologically different biomarker reflecting acute endogenous stress, such as copeptin, might allow for a rapid and accurate rule out of AMI already at initial presentation without serial blood sampling.” To do so, they enrolled 492 patients with symptoms suggestive of AMI in an ED in Switzerland and collected blood samples at presentation and after 3 h and 6–9 h, which were tested for troponin T and copeptin. They showed that copeptin levels were significantly higher in patients with AMI than in patients with other diagnoses, including unstable angina. Copeptin levels were particularly high in AMI-patients who tested negative for troponin T values at presentation and in patients who presented within 0–4 h after the onset of symptoms. With use of cutoff values of 0.01 μg/l or less for troponin T and less than 14 pmol/l for copeptin, the combined use of these two markers at presentation yielded a negative predictive value (NPV) for the diagnosis of non-ST-segment-elevation myocardial infarction (NSTEMI) of 99.7 %.

Keller et al. [8] confirmed these findings in a chest pain unit (CPU) population of 1,386 patients with chest pain or equivalent symptoms, where the marker combination at the 99th percentile cutoff for sensitive troponin I and 9.8 pmol/l for copeptin showed an NPV of 99 % (95 % confidence interval 96.6–99.9 %). Additionally, they reported the results of serial blood sampling in a subgroup of patients who presented within 2 h after symptom onset to illustrate the complementary kinetics of troponin T, the concentration of which increased 6 h after admission, and copeptin, the concentration of which decreased during the first 6 h after a peak at presentation, in patients with AMI.

Since then, many articles have been published, partly confirming and partly contradicting these findings. To be able to correctly judge these publications, a number of factors have to be considered.

## The Copeptin Cutoff

Keller et al. [8] evaluated different potential cutoff values for copeptin in a large reference population (*n* = 5,000), where the 99th percentile cutoff value was 18.9 pmol/l, the 97.5th percentile value was 13 pmol/l, and the 95th percentile value was9.8 pmol/l. Most clinical studies used a copeptin cutoff of 14 pmol/l. This cutoff was driven by the first copeptin assay available, which did not allow quantification of copeptin below this value [lower detection limit 4.8 pmol/l, functional assay sensitivity (lowest value with an interassay coefficient of variation below 20 %) below 12 pmol/l, limit of quantification 14.1 pmol/l] (manufacturer’s data). In 2011, an ultrasensitive copeptin assay was released, exhibiting a lower detection limit of less than 1 pmol/l, a functional assay sensitivity of less than 2 pmol/l, and a value for a coefficient of variation of 10 % of 2.5 pmol/l (manufacturer’s data). Mainly because 14 pmol/l was used in the first publications, this cutoff is still used in most studies. The lower the cutoff value though, the higher is the NPV for the diagnosis of AMI. To minimize the number of false-negative patients, a 95th percentile value (10 pmol/l) might be advisable. Table 2 shows a number of copeptin studies where different cutoff values were used, for comparison.

**Table 2:** Copeptin studies evaluating different copeptin cutoff values

Study	MI prevalence	Troponin assay	Copeptin assay	NPV^a^	Comments
Giannitsis et al. [9•]	CPU population (single-center study). AMI 27.0 % (136/503).NSTEMI 17.3 % (87/503)	hsTnT (Roche Diagnostics) <14 ng/l	Copeptin KRYPTOR (Thermo Fisher B·R·A·H·M·S) <14 pmol/l	hsTnT alone 95.8 % (92.6-97.9 %). hsTnT plus copeptin 98.6 % (95.8-99.7 %). NSTEMI only, hsTnT plus copeptin 99.03 % (96.6-99.9 %)	45.5 % of patients enrolled within 3 h after onset of symptoms; 19.5 % enrolled within 3–6 h after onset of symptoms
Potocki et al. [10•]	ED population (substudy of a multicenter study). AMI 15.7 % (184/1,170). Patients with preexisting CAD, AMI 18.0 % (78/433)	TnT (Roche Diagnostics, 4th generation) cutoff 0.01 μg/l. hsTnT (Roche Diagnostics) cutoff 14 ng/l	Copeptin KRYPTOR (Thermo Fisher B·R·A·H·M·S) 9 pmol/l	Patients with preexisting CAD, TnT alone 95 % (92.1-97.0 %), TnT plus copeptin 99.5 % (97.1-99.9 %), hsTnT alone 97.7 % (94.8-99.3 %), hsTnT plus copeptin 99.3 (96.3-99.9 %)	APACE substudy evaluating patients with preexisting CAD
Ray et al. [11•]	Pooled, selected ED population with history of CAD. AMI 8.0 % (36/451), NSTEMI 6.7 % (30/451)	2 EDs cTnI (Siemens Healthcare), 1 ED cTnI (Abbott Laboratories). Cutoffs below threshold of 10 % CV	Copeptin KRYPTOR (Thermo Fisher B·R·A·H·M·S)		Subanalysis from 3 prospective trials (Basel, Paris, and Toulouse) evaluating patients with a history of CAD
			>9.3 pmol/l	98 % (95-99 %)	
			>9.8 pmol/l	98 % (95-99 %)	
			>14.1 pmol/l	97 % (94-98 %)	
			>18.9 pmol/l	97 % (94-98 %) (all for NSTEMI)	
Charpentier et al. [12•]	ED population (single center). NSTEMI 14.8 % (95/641)	cTnI ADVIA Centaur (Siemens Diagnostics) >0.1 μg/l	Copeptin KRYPTOR (Thermo Fisher B·R·A·H·M·S)	cTnI alone 92.8 % (90.8-94.8 %).	Subanalysis of a single-center prospective study. STEMI excluded
				Combination:	
			<12 pmol/l	97.6 % (96.4-98.7 %)	
			<14 pmol/l	97.1 % (95.7-98.4 %)	
Charpentier [13•]	ED population (single center), NSTEMI 14.8 % (87/587). Fewer patients than in [12•] owing to insufficient blood samples	Sensitive TnI-Ultra ADVIA Centaur (Siemens Healthcare) cutoff 0.05 μg/l	Copeptin KRYPTOR (Thermo Fisher B·R·A·H·M·S) <12 pmol/l	Sensitive TnI-Ultra alone 94.9 % (92.6-96.6 %), sensitive TnI plus copeptin 99.1 % (97.4-99.8 %). 46.8 % of patients with low TIMI score, NPV 100 % (97.7-100 %) for biomarker combination	Subanalysis of a single-center prospective serum-bank study (same study as Charpentier et al. [12•]). STEMI excluded
Llorens et al. [14•]	ED population (multicenter, 28 sites) with probable ACS. NSTEMI 10.5 % (107/1,018)	Respective troponin of daily practice (23 EDs TnT) (0.03 ng/ml), 2 EDs hsTnT (0.013 ng/ml), 3 EDs TnI (0.04 ng/ml)	Copeptin KRYPTOR (Thermo Fisher B·R·A·H·M·S)	Copeptin only in troponin-negative patients:	Multipurpose study. COPED substudy: STEMI patients, patients who tested positive for troponin at admission, and patients with noncoronary chest pain excluded
			5 pmol/l 10 pmol/l 14 pmol/l 18 pmol/l	95 % 94.8 % 94.2 % 93.7 %	
Collinson et al. [15•]	ED population (multicenter study, 6 sites). NSTEMI 8.0 % (68/850)	Different TnT and TnI assays	Assay not reported. Cutoff 7.4 mg/l (not comparable with KRYPTOR results)	cTnI alone 98 % (0.97-0.99), cTnI plus copeptin 0.99 (0.97-1.0), cTnT alone 98 % (0.97-0.99), cTnT plus copeptin 98 % (0.97-0.99)	Subanalysis of the RATPAC study comparing troponin POCT with conventional management. High-risk patients and STEMI patients excluded

*ACS* acute coronary syndrome, *CAD* coronary artery disease, *CV* coefficient of variation, *cTnI* cardiac troponin I, *cTnT* cardiac troponin T, *hsTnT* high-sensitivity troponin T, *POCT* point-of-care testing, *STEMI* ST-segment-elevation myocardial infarction *TIMI* thrombosis in myocardial infarction

^a^NPV for marker combination if not indicated otherwise.

## AMI Prevalence and Pretest Probability

Although sensitivity and specificity are independent of the prevalence of the disease, both the positive predictive value and the NPV change with different disease prevalence. The NPV decreases with increasing prevalence, whereas the positive predictive value increases. In most rule-out studies on copeptin and troponin, the NPV is the primary measure of interest, determining success or failure of the new concept. Table 1 provides a list of studies with their respective AMI prevalence and the NPV calculated for this population.

Even though the data are difficult to compare for the multitude of factors influencing the study results, the data reflect a relatively low NPV in cohorts with an AMI prevalence above 20 % (Table 3).

**Table 3:** Copeptin studies in populations with high MI prevalence

Study	MI prevalence	Troponin assay	Copeptin assay	NPV^a^	Comments
Afzali et al. [16•]	CPU population (single center). AMI 46.5 %(107/230), NSTEMI 36.1 % (83/230)	TnI-Ultra (Siemens Healthcare) cutoff <0.04 ng/ml (99th percentile)	Copeptin KRYPTOR (Thermo Fisher B·R·A·H·M·S) <14 pmol/l	97.3 %	13 % of patients with a GRACE score greater than 140. Onset of symptoms after more than 12 h in 37.8 % of patients
Sukul et al. [17•]	Single-center study, setting not reported. AMI 25.7 % (104/405), NSTEMI 22.4 % (91/405)	Local cTnI (Centaur, Siemens Healthcare)cutoff 100 ng/l. Sensitive cTnI (TnI-Ultra, Siemens Healthcare) cutoff 40 ng/l (99th percentile)	Copeptin KRYPTOR (Thermo Fisher B·R·A·H·M·S) 14 pmol/l	cTnI alone 92 % (89-95 %), sensitive TnI alone 98 % (95-99 %), sensitive TnI plus copeptin 97 % (94-99 %) (for all AMI). In early presenters (<6 h), sensitive TnI 100 % (96-100 %), sensitive TnI plus copeptin 100 % (95-100 %)	No analysis of NSTEMI patients only
Eggers et al. [18•]	CPU population, NSTEMI 35.6 % (128/360) FAST II: 2000–2001 FASTER I: 2002-2003	hsTnT (Roche Diagnostics) 14 ng/l. NSTEMI diagnosis based on routine TnI result (Stratus CS, Siemens Healthcare)	Ultrasensitive copeptin KRYPTOR PLUS (Thermo Fisher B·R·A·H·M·S) >14 pmol/l	hsTnT alone 86.5 % (81.0-90.0 %),hsTnT plus copeptin 89 % (83.1-93.3 %)	Pooled population of patients included in the FAST II and FASTER I studies with available results for biomarkers, only NSTEMI and symptom onset <8 h

*GRACE* Global Registry of Acute Coronary Events

^a^NPV for marker combination if not indicated otherwise.

For the same reason, a test can exhibit very different predictive values when it is administered to patients at different levels of risk (Table 4). Again, in a high-risk population the NPV is lower, whereas it is higher in a low-risk population. The marker combination of troponin and copeptin has been shown to achieve the best results in patients at low- to intermediate risk of AMI. Bohyn et al. [19••] tested a rule-out strategy using copeptin, troponin, and the Global Registry of Acute Coronary Events (GRACE) score. Here, both markers had to test negative and the GRACE score had to be below 108 points. With this concept, the NPV was 99 % (95 % confidence interval 94–100 %).

**Table 4:** Copeptin studies including pretest probability (*PTP*)

Study	MI prevalence	Troponin assay	Copeptin assay	NPV^a^	Comments
Chenevier-Gobeaux et al. [20••]	ED population (3 centers). AMI 14.2 % (45/317), NSTEMI 10.1 % (32/317)	2 EDs TnI (Siemens Healthcare) >0.14 μg/l, 1 ED cTnI (Beckman Coulter) >0.06 μg/l	Copeptin KRYPTOR (Thermo Fisher B·R·A·H·M·S) ROC-optimized cutoff 10.7 pmol/l	cTnI alone 95 % (92-97 %), cTnI plus copeptin 99 % (97-100 %) (in low-PTP group 100 %)	Presentation within 3 h after onset of symptoms in 61 % of patients. 47 % of patients with low PTP; 37 % of patients with moderate PTP
Bohyn et al. [19••]	ED population (health center group/coronary care unit network). NSTEMI 15.9 % (39/245)	hsTnT (Roche Diagnostics) 14 ng/l	Copeptin KRYPTOR (Thermo Fisher B·R·A·H·M·S) 14 pmol/l	hsTnT alone 92 % (88–95), hsTnT plus copeptin 95 % (90–98), hsTnT plus copeptin plus GRACE score <108, 99 % (94-100 %)	Combination of hsTnT, copeptin, and GRACE score
Maisel et al. [22••]	ED population (multicenter with 16 sites). AMI 7.9 % (156/1,967), NSTEMI 5.9 % (116/1,967)	cTnI (TnI-Ultra ADVIA Centaur, (Siemens Healthcare) <40 ng/l (99th percentile). Local site biomarker for diagnosis	Copeptin KRYPTOR (Thermo Fisher B·R·A·H·M·S) <14 pmol/l	Troponin alone 98.8 %, troponin plus copeptin 99.2 % (98.5-99.6 %). In patients with low AMI likelihood, NPV 99.8 %; in patients with intermediate AMI likelihood, NPV 99.6 %	VAS score for likelihood of ACS and AMI as judged by ED physician before and after troponin test result

*PTP* pretest probability, *ROC* receiver operating characteristic, *VAS* visual analogue scale

^a^NPV for marker combination if not indicated otherwise

Two studies have shown that the pretest probability can also be determined by the judgment of the treating physician.

Chenevier-Gobeaux et al. [20••] divided their cohort into three pre-test-probability groups (low, medium, or high), as assessed by the treating ED physician after the first clinical evaluation but before the biomarker results were available. The NPVs were 100 (78-100)% in high-risk patients, 98 (87-100)% in medium-risk patients, and 100 (95-100)% in low-risk patients. In the CHOPIN study [22••], the treating ED physicians were asked to judge the likelihood of ACS and AMI on a visual analogue scale. The NPV for patients with intermediate risk was 99.6 % and for patients with low risk was 99.8 %;the NPV for the entire cohort was 99.2 %.

## Time Point of Blood Sampling

The concentration of copeptin is known to increase with the acute event and to then decrease rapidly to normal values within hours [8]. Thus, the time point of copeptin testing is crucial for this concept. In studies with most patients presenting late after the onset of symptoms, copeptin testing is unlikely to provide added value to troponin testing, as most patients will already be troponin-positive at admission to the ED or CPU [23•]. This was taken to an extreme in a study by Karakas et al. [24•] where copeptin was measured at a median of 4.3 h after presentation to the ED in a study which was primarily set up to evaluate CT angiography in patients with suspected ACS.

It is important to note that in late presenters the new strategy of a single copeptin–troponin measurement at presentation does not harm the patient, as the concentration of troponin will at this stage be increased in patients with NSTEMI and discharge of false-negative patients is thus unlikely.

## Study End Point

Some studies have evaluated the diagnostic performance of copeptin and troponin for the diagnosis of ACS rather than the diagnosis of AMI [24•, 25•, 26•]. Reichlin et al. [1] clearly showed that the concentration of copeptin is not increased in patients with unstable angina.

The combined marker strategy also seems to perform better in cohorts with NSTEMI patients as compared with all AMIs [27•]. Nevertheless, given that the diagnosis in ST-segment-elevation myocardial infarction (STEMI) is based on ECG rather than biomarker results, STEMI patients have often been excluded when the diagnostic performance of these markers has been analyzed. Specifically, in unclear situations when patients present early, there is also a potential benefit of copeptin testing for patients who are finally categorized as having STEMI.

## Application of the New Strategy in Clinical Practice

All the aforementioned studies are observational studies with retrospective copeptin measurement, where the copeptin value did not change patient care. Recently, the BIC-8 study, the first interventional, randomized process trial, evaluating the early-rule-out strategy in clinical practice, has been published [28••]. Low- to intermediate-risk patients with suspected ACS (*n* = 902) were randomized into either the standard group, receiving standard diagnostic workup and care, or the copeptin group, where further care depended on the copeptin value. In this group, copeptin-positive patients were considered higher risk and were admitted for standard workup, whereas copeptin-negative patients were considered low risk and were discharged to ambulant care, including a visit with a resident cardiologist within three working days. Importantly, the ultimate decision to discharge or admit a patient was left to the discretion of the treating physician on the basis on his/her clinical workup.

In this multicenter, international study, the major adverse cardiac event proportion at 30 days was not higher in the copeptin group (5.17 %) as compared with the standard group (5.19 %), suggesting safety comparable to that of the current standard process. Secondary end point analysis showed that patients in the copeptin group were discharged directly from the ED/CPU more often (67.6 % in the copeptin group vs 12 % in the standard group) and earlier (median length of stay for patients with AMI exclusion 4 h in the copeptin group and 7 h in the standard group), suggesting an effectiveness benefit of the new process.

## Conclusion

There is ample evidence that combined testing of copeptin and troponin at presentation in low- to intermediate-risk patients with suspected ACS to rule out NSTEMI is a promising strategy. From a review of publications on this new concept, all the aforementioned factors—copeptin cutoff, pretest probability, and time point of copeptin testing—need to be considered to be able to judge the results appropriately.

When applying the strategy in clinical practice, physicians need to be aware that copeptin–troponin rule-out should be applied only in patients at low-to intermediate risk of ACS who are generally fit to be discharged. It is important to keep in mind that biomarkers, like all diagnostic tests, need to be applied with an appropriate objective, on the basis of a thorough clinical workup to be able to interpret the results correctly.

**Fig. 1:**
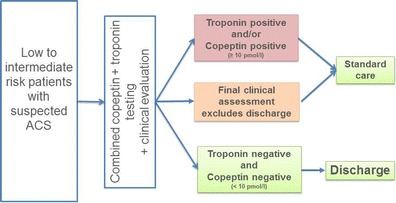
Suggested new process for the workup of low- to intermediate-risk patients with suspected acute coronary syndrome (*ACS*) using an early rule-out strategy with combined troponin and copeptin testing

BIC-8 has indicated that low-to intermediate-risk patients with a negative copeptin–troponin marker combination can be safely discharged. Figure 1 shows a flowchart for the suggested new process of an ACS workup. Still, clinical process studies are faced with a number of issues limiting the evaluation of a single step in a network of influencing factors and decisions. Thus, the results of this trial should be confirmed in further interventional trials. If the process is implemented in clinical practice, outcomes of patients managed with the new process strategy should be monitored closely in clinical registries to be able to judge the real-life safety and effectiveness.
